# Frequent mutations of *KRAS* in addition to *BRAF* in colorectal serrated adenocarcinoma

**DOI:** 10.1111/j.1365-2559.2011.03821.x

**Published:** 2011-04

**Authors:** Karoliina Stefanius, Laura Ylitalo, Anne Tuomisto, Rami Kuivila, Tiina Kantola, Päivi Sirniö, Tuomo J Karttunen, Markus J Mäkinen

**Affiliations:** Department of Pathology, Institute of Diagnostics, University of OuluOulu, Finland

**Keywords:** *BRAF*, colorectal cancer, DNA hypermethylation, h*MLH1*, *KRAS*, *MGMT*, microsatellite instability, serrated adenocarcinoma

## Abstract

**Aims:**

To define the occurrence of *KRAS* and *BRAF* mutations, microsatellite instability (MSI), and *MGMT* and h*MLH1* methylation and expression in colorectal serrated adenocarcinoma.

**Methods and results:**

*KRAS* codon 12/13 and 59/61 and *BRAF* V600E mutations, MSI, and *MGMT* and h*MLH1* methylation and expression in 42 serrated adenocarcinomas and 17 serrated adenomas were compared with those in 59 non-serrated colorectal carcinomas (CRCs) and nine adenomas. *KRAS* and *BRAF* mutations were observed in 45% and 33% of serrated adenocarcinomas and in 27% and 0% of non-serrated CRCs (*P* < 0.001). The *KRAS* c12G→A transition was the predominant type of mutation in serrated adenocarcinomas. Forty-two per cent of *BRAF*-mutated serrated adenocarcinomas showed high-level MSI (MSI-H) (*P* = 0.075), 100% showed h*MLH1* methylation (*P* = 0.001) and 90.9% showed *MGMT* methylation (*P* = 0.019). Fifty-six per cent of serrated adenocarcinomas with microsatellite stability/low-level microsatellite instability harboured *KRAS* mutations. In non-serrated cancers, *KRAS* mutations were not associated with MSI status.

**Conclusions:**

A high combined mutation rate (79–82%) of *KRAS* and *BRAF* in serrated adenomas and adenocarcinomas indicates that mitogen-activated protein kinase activation is a crucial part of the serrated pathway. *BRAF* mutations are specific for serrated adenocarcinoma and identify a subset of serrated adenocarcinomas with gene methylation and a tendency for MSI-H. A high frequency of *KRAS* mutations in serrated adenocarcinomas suggests that a significant proportion of *KRAS*-mutated CRCs originate from serrated precursors, thus challenging the traditional model of Vogelstein.

## Introduction

Colorectal cancer (CRC) is the second most common cancer type in the Western world.^1^ For a long time, non-serrated adenomas were thought to represent the only significant precursor lesion for CRC.^2^ However, it is now apparent that the development of 15–20% of sporadic CRCs is not explained by Vogelstein's adenoma–carcinoma model. These cancers often show concurrent *BRAF* mutations and DNA CpG island hypermethylation (CIM), and associate with high-level DNA microsatellite instability (MSI-H) via methylation of the DNA mismatch repair gene h*MLH*.^3^,^4^ It is generally believed that these cancers originate from serrated polyps, because this combination of alterations is frequent in serrated polyps (hyperplastic polyps, sessile serrated adenomas, mixed hyperplastic/adenomatous polyps, also known as admixed polyps, and traditional serrated adenomas), but absent in sporadic non-serrated adenomas. The serrated pathway has emerged as the second most significant pathway leading to CRC. A smaller subset of CRCs, at least 7.5%, can be distinguished by their morphology as being derived from serrated precursor lesions, even when such precursor lesions are no longer visible. We have referred to them as ‘serrated adenocarcinoma’ to indicate their origin in serrated polyps. Although many of these cancers retain a serrated pattern of epithelium, poorly differentiated ones are better characterized by abundant eosinophilic cytoplasm and a trabecular growth pattern.^4^,^5^

*KRAS* mutations have been considered to be the hallmark mutations of Vogelstein's adenoma–carcinoma model. Given the fact that the development of CRCs from serrated polyps with *KRAS* mutations has not yet been described, *KRAS*-mutated serrated polyps have been suggested to make a minor contribution to CRC development.^6^,^7^

KRAS and BAF belong to the intracellular RAS*/*RAF/MEK/mitogen-activated protein kinase (MAPK) cascade, which mediates cellular responses to growth signals. Activating mutations of *KRAS* occur in 30–50% of CRCs.^8^,^9^ Most (90%) are found in codons 12 and 13 of exon 1, and about 5% in codons 59 and 61 of exon 2.^10^ A single missense mutation of *BRAF* (*BRAF* V600E) accounts for 80% of the mutations in CRCs.^11^,^12^ Both *BRAF* and *KRAS* mutations have been found in the earliest detectable lesions with a serrated morphology, i.e. in hyperplastic/heteroplastic aberrant crypt foci.^4^,^13^*BRAF* mutations have been reported in 19–36% of hyperplastic polyps, in 40–89% of admixed polyps, in 75–82% of sessile serrated adenomas, and in 20–66% of traditional serrated adenomas.^3^,^4^,^14^,^15^ Similarly, *KRAS* mutations have been reported in about 18% of aberrant crypt foci, in 4–37% of hyperplastic polyps, in 60% of admixed polyps, in up to 80% of traditional serrated adenomas, and in up to 10% of sessile serrated adenomas.^4^,^16^

A recent study based on 11 cases found *BRAF* mutations to be frequent and highly specific for serrated adenocarcinoma,^17^ but the significance of *KRAS* mutations in serrated adenocarcinoma development is not known. Therefore, this study was conducted in order to identify the prevalence of *BRAF* and *KRAS* mutations in serrated adenomas and serrated adenocarcinomas, and their potential associations with both the microsatellite instability (MSI) status and the methylation of h*MLH1* and *MGMT*, which are known to be altered in serrated polyps and in the sporadic MSI/methylator pathway to CRC.

## Materials and methods

### Materials

**Figure 1:**
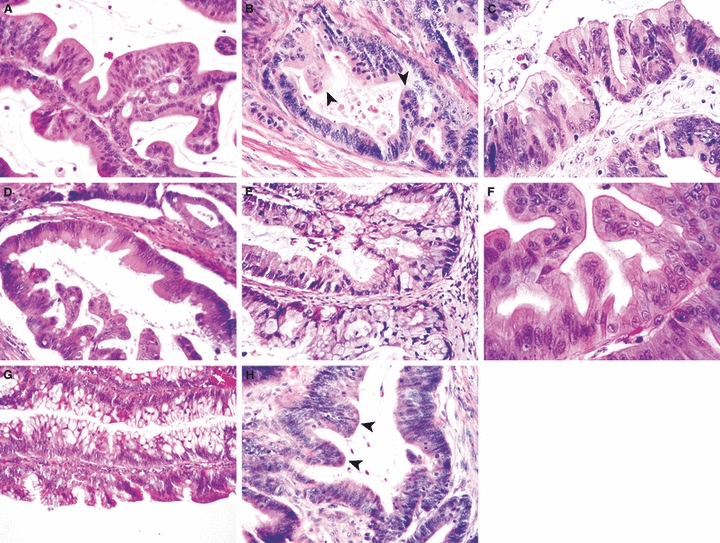
Representative histological sections of serrated adenocarcinomas in regard to *KRAS* or *BRAF* mutations and microsatellite instability (MSI) status. **A**–**C**, Cases of serrated adenocarcinomas with *BRAF V600E* mutations. **D**–**F**, Serrated adenocarcinomas with *KRAS* c12 mutations. **G**–**H**, Serrated adenoma and adenocarcinoma wild type for *BRAF* and *KRAS*, but presenting with high-level MSI (MSI-H). **A**, A case of serrated adenocarcinoma of the proximal colon with MSI-H, and harbouring a *BRAF V600E* mutation. This case is well differentiated and mucin-producing. The serrated epithelial folding is conspicuous. **B**, Another case of serrated adenocarcinoma of the proximal colon with MSI-H, and harbouring a *BRAF V600E* mutation, initially diagnosed as conventional adenocarcinoma. Serrated epithelial folding is less conspicuous, but present (arrowheads). **C**, A case of a serrated adenocarcinoma of the proximal colon with microsatellite stability (MSS), presenting with *BRAF V600E* mutation. **D**, A case of a serrated adenocarcinoma of the proximal colon with MSS, and harbouring a *KRAS* mutation. The serrations are apparent. **E**, A case of serrated adenocarcinoma of the proximal colon with MSS/low-level MSI, and harbouring a *KRAS* mutation. There is abundant clear cytoplasm, preserved polarity and luminal serration. **F**, A case of serrated adenocarcinoma of the proximal colon with MSI-H, and harbouring a *KRAS* mutation. There is a prominent serrated pattern with epithelial tufting towards the lumen. **G**, A case of traditional serrated adenoma of the proximal colon. This tumour was wild type for both *KRAS* and *BRAF*, and had MSI-H. The patient was 78 years old. There was no evidence of hereditary non-polyposis colorectal carcinoma. **H**, A case of serrated adenocarcinoma adjacent to the traditional serrated adenoma shown in (**G**). The tumour shows preserved serrations (arrowheads).

Altogether, 47 serrated adenocarcinomas were obtained for this study. The histology was confirmed by two independent pathologists (M.J.M. and T.J.K.) from the haematoxylin and eosin-stained slides. Samples were diagnosed as serrated adenocarcinomas when the cancer tissue was composed of epithelial proliferation reminiscent of serrated adenoma, i.e. the cytoplasm of these cells was clear or eosinophilic, and when the cellular changes were accompanied by a serrated growth pattern, i.e. cells forming pseudopapillary or pseudocribriform structures with tufting of the cells into the lumen without true papillary structures with a fibrovascular core.^5^ CRC specimens with serrated morphology were screened from the previously described, consecutively collected and population-based Finnish collection of 466 samples.^18^ This material yielded 38 serrated cases, of which 35 were previously diagnosed as serrated cancers.^5^ After careful review, three cases were reclassified as serrated adenocarcinomas. These cases did not have a residual serrated adenoma component (see Figure 1B for an example). The original series of samples was extended to cover the years 1997–1998, raising the number of samples in the consecutive series to 552 and the number of serrated CRCs to 45. Two additional serrated cases removed between the years 2006 and 2007 were included. With five serrated cases of the consecutive series lacking sufficient sample material, 42/47 serrated CRCs were included in the DNA analyses of the present study. For the sake of simplicity, we have referred to all the other adenomas and carcinomas as ‘non-serrated’ in this paper.

A control series of non-serrated adenocarcinomas (*n* = 32) was selected from the demographic series of 552 cases to match gender, location, Dukes’ stage and World Health Organization histological grade. In the proximal colon, serrated adenocarcinomas represented such a large proportion that matched controls could not be obtained for all cases. Therefore, the matched control group remained smaller than the study group, and thus a set of 27 unmatched non-serrated adenocarcinomas selected from the same series to the study was added, yielding a total of 59 non-serrated adenocarcinomas.

Residual benign, serrated adenoma in contact with cancer tissue was present in 28/42 serrated adenocarcinoma cases. For purposes of comparison, adenoma tissue was separately collected for the analyses. In nine cases of serrated adenocarcinoma, the residual serrated adenoma tissue was available for the analyses. An additional set of 17 serrated adenomas and nine non-serrated adenomas with a size of 5 mm or more was retrieved from the archives of Oulu University Hospital Department of Pathology.

### DNA extraction and MSI analysis

DNA extraction and MSI analysis were performed as described previously.^5^,^19^ Tumour DNA was analysed with the NIH consensus marker panel, and distinction of MSI-H from microsatellite stability (MSS)/low-level MSI (MSI-L) was made according to the NIH consensus statement.^20^

### Methylation analysis

Methylation analyses of the promoter sequences of the h*MLH1* and *MGMT* genes were performed using the methylation-specific polymerase chain reaction (PCR)^21^ based on bisulphite pretreatment of DNA. The primer pairs used for the methylated and unmethylated templates of h*MLH1* were TTTTTTAGGAGTGAAGGAGGTTACG (forward) and GCCACTACGAAACTAAACACGAA (reverse), and TTTTTAGGAGTGAAGGAGGTTATGG (forward) and AAACACCACTACAAAACTAAACACAAA (reverse), respectively. The primer pairs used for the methylated and unmethylated templates of *MGMT* were TTTCGACGTTCGTAGGTTTTCGC (forward) and GCACTCTTCCGAAAACGAAACG (reverse),^22^ and TTTGTGTTTTGATGTTTGTAGGTTTTTGT (forward) and AACTCCACACTCTTCCAAAAACAAAACA (reverse), respectively. Bisulphite-treated genomic DNA (100 ng) was used as a template in the PCR. The 2× JumpStart REDTaq ReadyMix (Sigma-Aldrich, St Louis, MO, USA) was used according to the manufacturer's instructions. Commercial methylated DNA (CpGENOME Methylated DNA) and unmethylated DNA (CpGENOME Unmethylated DNA) were included in all the analyses as internal controls (Chemicon International, Temecula, CA, USA). The PCR products were visualized with UV illumination on a 2.5% agarose gel. The results of the methylation analyses of the h*MLH1* and *MGMT* genes were compared with the intensity of the immunoreaction of the corresponding proteins.^5^

### Mutation analysis

Mutation analyses of *BRAF* V600E and *KRAS* were performed by direct sequencing with PCR-amplified template DNA (25 ng), using a dynazyme DNA polymerase kit (Finnzymes Oy, Espoo, Finland). The primers used in the analysis were AAACTCTTCATAATGCTTGCTCTG (forward) and GGCCAAAAATTTAATCAGTGGA (reverse) for *BRAF* V600E, TGGTGGAGTATTTGATAGTGTA (forward) and ATGGTCCTGCACCAGTAATA (reverse) for *KRAS* c12/13, and TGAAGTAAAAGGTGCACTGTAATA (forward) and TAAACCCACCTATAATGGTGAA (reverse) for *KRAS* c59/61. PCR conditions are available on request. The amplifications were performed with a PTC-200 thermal cycler (MJ Research, Waltham, MA, USA). The PCR products were enzymatically purified with EXOSAPit (USB, Cleveland, OH, USA) according to the manufacturer's instructions. Sequencing of the product was performed in both directions on the ABI 3130*xl* Genetic Analyzer (Applied Biosystems, Foster City, CA, USA), with the forward and reverse primers. The data obtained were analysed with CHROMAS 1.6 sequencing analysis software (Technelysium Pty, Halensvale, Australia). All mutations were reconfirmed by independent PCR reactions and sequencing.

### Immunohistochemistry

MLH1 and MSH2 analyses were carried out as described previously.^5^ Briefly, mouse monoclonal antibodies for MLH1 and MSH2 (BD-PharMingen, San Diego, CA, USA) were applied at a dilution of 1:25 (MLH1) or 1:50 (MSH2) for 1 h at room temperature. The reaction in the tumour area was considered to be negative if there was no staining in any of the tumour cell nuclei. For MGMT analysis, tissue sections were pretreated in 0.01 m citrate (pH 6.0) buffer in a microwave oven at 800 W for 2 min and at 300 W for 10 min. Primary antibody for MGMT was applied at a dilution of 1:300 for 1 h at room temperature. A Dako EnVision kit (Dako, Copenhagen, Denmark) was used in the detection of the bound antibodies, with 3,3′-diaminobenzidine as a chromogen. The reaction in the tumour area was considered to be negative if less than 10% of the tumour cell nuclei stained positive.

### Statistical analysis

Tests were performed with statistical software (SPSS 16.1; SPSS, Chicago, IL, USA). The chi-square-test or Fisher's exact test were used unless otherwise stated. A *P*-value of <0.05 was considered to be statistically significant.

### Ethical aspects

The protocol (58/2002) was approved by the Ethical Committee of Oulu University Hospital.

## Results

The success rates of each DNA analyses are shown in Table 1. Clinicopathological features of the study material are presented in Tables 2 and 3, and examples of serrated adenocarcinomas in relation to *KRAS* and *BRAF* mutations and MSI status are illustrated in Figure 1. *BRAF* and *KRAS* mutations did not coexist. The prevalence and distribution patterns of *BRAF* V600E, *KRAS* c12/13 and *KRAS* c59/61 mutations are presented in Table 4. *BRAF* mutations were frequent (33.3%; 14/42) and specific to serrated adenocarcinomas. After a careful histopathological re-evaluation of the cases, three *BRAF*-mutated cases, originally placed in the control group, were reclassified as serrated adenocarcinomas because the serrated morphology was preserved in the tumour. *KRAS* mutations were more frequent in serrated adenocarcinomas (45.2%) than in non-serrated adenocarcinomas (27.1%; *P* = 0.002). The higher frequency of *KRAS* mutations was clearly more evident (although not statistically significant) in cancers with a residual serrated adenoma (57.1%, 16/28), being observed twice as often as *BRAF* mutations (28.6%, 8/28). The combined prevalence of *BRAF* and *KRAS* mutations (78.6%) in serrated adenocarcinomas was higher than in non-serrated adenocarcinomas (27.1%; *P* < 0.001; Table 4), and the combined prevalence of *BRAF* and *KRAS* mutations in serrated adenocarcinomas with a residual serrated adenoma component reached 85.7% (24/28). In adenomas, *BRAF* mutations were specific to serrated adenomas, as none of the non-serrated adenomas showed a *BRAF* mutation (*P* = 0.058). Only one non-serrated adenoma carried a *KRAS* mutation. Either *BRAF* or *KRAS* mutation was observed in 82.4% of serrated adenomas (*P* < 0.001; Table 4).

**Table 1:** The success rates of DNA analyses

	Serrated CRCs	Matched non-serrated CRCs	All non-serrated CRCs	Serrated adenomas	Serrated adenomas adjacent to cancer	Non-serrated adenomas
*BRAF* V600E	42/42	31/32	49/59	17/17	9/9	9/9

*KRAS* c12/13	42/42	32/32	45/59	17/17	9/9	9/9

*KRAS* c59/61	40/42	31/32	56/59	17/17	8/9	9/9

MSI analyses	37/42	30/32	56/59	11/17	2/9	4/9

Methylation analyses						
h*MLH1*	27/42	29/32	51/59	–	–	–

*MGMT*	29/42	29/32	53/59	–	–	–

CRC, Colorectal carcinoma.

**Table 2:** The clinical and pathological features of serrated and non-serrated adenocarcinomas

	Serrated adenocarcinomas (*n* = 42)	Non-serrated adenocarcinomas (*n* = 59)	*P*-value (*χ*^2^-test)
Mean age in years (range)	67.5 (43–85)	68.5 (38–88)	

Gender, *N* (%)			
Male	17 (40.5)	23 (39.0)	0.880
	
Female	25 (59.5)	36 (61.0)	

Location, *N* (%)			
Proximal colon	24 (57.1)	20 (33.9)	0.050
	
Distal colon	6 (14.3)	9 (15.3)	
	
Rectum/rectosigmoid colon	12 (28.6)	30 (50.8)	

Grade, *N* (%)			
I	14 (33.3)	11 (18.6)	0.085
	
II	20 (47.6)	41 (69.5)	
	
III	8 (19.0)	7 (11.9)	

Dukes' stage, *N* (%)			
A	6 (14.3)	13 (22.0)	0.699
	
B	20 (47.6)	23 (39.0)	
	
C	10 (23.8)	16 (27.1)	
	
D	6 (14.3)	7 (11.9)	

Mucinous, *N* (%)			
No	26 (61.9)	54 (91.5)	<0.001
	
Yes	16 (38.1)	5 (8.5)	

MSI			
MSI-H	7 (18.9)	4 (7.1)	0.164
	
MSS/MSI-L	30 (81.1)	52 (92.9)	

MSI, Microsatellite instability; MSI-H, high-level MSI; MSI-L, low-level MSI.

**Table 3:** The clinical and pathological features of serrated and non-serrated adenomas

	Serrated adenomas (*n* = 26)*	Non-serrated adenomas (*n* = 9)	*P*-value (*χ*^2^-test)
Mean age in years (range)	64.1 (36–84)*	74.1 (60–83)	

Gender, *N* (%)			
Male	13 (50.0)	8 (90)	0.040
	
Female	13 (50.0)	1 (10)	

Location, *N* (%)			
Proximal colon	6 (20)	0 (0)	0.396
	
Distal colon	4 (5)	2 (20)	
	
Rectum/rectosigmoid colon	16 (75)	7 (80)	

Dysplasia, *N* (%)			
Mild	6 (35)	3 (40)	0.024
	
Moderate	8 (25)	6 (60)	
	
Severe	12 (40)	0 (0)	

* All serrated adenomas, including adenomas adjacent to cancer (*n* = 9).

**Table 4:** The prevalence and distribution of *BRAF* V600E and *KRAS* (codons 12/13 and 59/61) mutations according to the type of neoplasm

	*BRAFV* 600E	*KRAS* (all)	*KRAS* c12/13	*KRAS* c59/61	Either *BRAF* or *KRAS*
*Serrated adenocarcinomas (n = 42)*					

No adjacent adenoma component (*n* = 14)					
With mutation/all	6/14	3/14	2/14	1/14	9/14

%	42.9	21.4	14.3	7.1	64.3

With adjacent serrated adenoma (*n* = 28)					
With mutation/all	8/28	16/28	15/28	1/28	24/28

%	28.6	57.1	53.6	3.6	85.7

All (*n* = 42)					
With mutation/all	14/42	19/42	17/42	2/42	33/42

%	33.3	45.2	40.5	4.8	78.6

*Matched non-serrated carcinomas (n = 32)*					
With mutation/all	0/32	13/32	12/32	1/32	13/32

%	0	40.6	37.5	3.1	40.6

*P*	<0.001	0.894	1.00	0.842	<0.001

*All non-serrated carcinomas (n = 59)*					
With mutation/all	0/59	16/59	15/59	1/59	16/59

%	0	27.1	25.4	1.7	27.1

*P*	<0.001	0.002	0.002	0.848	<0.001

*Serrated adenomas (n = 17)*					
With mutation/all	7/17	7/17	6/17	1/17	14/17

%	41.2	41.2	35.3	5.9	82.4

*Non-serrated adenomas (n = 9)*					
With mutation/all	0/9	1/9	1/9	0/9	1/9

%	0	11.1	11.1	0	11.1

*P*	0.058	0.243	0.453	1	<0.001

*Serrated adenomas adjacent to cancer (n = 9)*					
With mutation/all	2/9	4/9	3/9	1/9	6/9

%	22.2	44.4	33.3	11.1	66.7

*P*	0.068	0.353	0.6	0.436	0.001

Among non-serrated adenocarcinomas, *KRAS* c12/13 and c59 mutations were found in 16 cases (Table 4). The mutation pattern of *KRAS* c12/13 and c59/61 is shown in Table 5. The c12 G→A transitions were found in 52.6% (10/19) of *KRAS*-mutated serrated adenocarcinomas and in 12.5% (2/16; *P* = 0.047) of *KRAS*-mutated non-serrated cancers. This transition showed a distinct association with serrated adenocarcinomas, being present in 24% of cases (10/42), but in only 3.4% of non-serrated carcinomas (2/59; *P* = 0.001; Fisher's exact test).

**Table 5:** The observed sequence changes and the corresponding amino acid substitutions in *KRAS* c12/13 and c59/61 in the study population

KRAS c12/13 and c59/61 sequence change	Amino acid change	Serrated adenocarcinomas, *N* (%)	Non-serrated adenocarcinomas, *N* (%)	Total, *N* (%)	*P*-value (Fisher's exact test)	Serrated adenomas	Non-serrated adenomas	Total	*P*-value (Fisher's exact test)
c12 GGT→GTT	Gly→Val	4 (21.1)	7 (43.8)	11 (31.4)	0.047	4 (36.4)	0 (0)	4 (33.3)	1.00
			
c12 GGT→TGT	Gly→Cys	1 (5.3)	3 (18.8)	4 (11.4)		0 (0)	0 (0)	0 (0)	
			
c12 GGT→GAT	Gly→Asp	10 (52.6)	2 (12.5)	12 (34.3)		4 (36.4)	1 (100)	5 (41.7)	
			
c13 GGC→GAC	Gly→Asp	2 (10.5)	3 (18.8)	5 (14.3)		1 (9.1)	0 (0)	1 (8.3)	
			
c59 GCA→GGA	Ala→Gly	0 (0)	1 (6.3)	1 (2.9)		0 (0)	0 (0)	0 (0)	
			
c61 CAA→AAA	Gln→Lys	2 (10.5)	0 (0)	2 (5.7)		2 (18.2)	0 (0)	2 (16.7)	
			
Total		19 (100)	16 (100)	35 (100)		11 (100)	1 (100)	12 (100)	

The MSI analyses in carcinoma material were successful in 93 cases (Table 1). Concurrent data from the MSI analyses and the *KRAS*/*BRAF* mutation analyses were obtained in 74 carcinoma cases (Table 6). Five *BRAF* mutations and one *KRAS* c61 mutation were observed among 11 MSI-H cancers when unmatched cases were included (*P* = 0.007) (Table 6). Serrated adenocarcinomas presenting with MSI-H were unlikely to be hereditary non-polyposis CRC cases. They occurred in old patients (Table 7) and presented with an adjacent sessile serrated adenoma (cases 1 and 5 in Table 7) or traditional serrated adenoma (cases 2, 3 and 6 in Table 7). Sixty-three MSS/MSI-L cancers showed an almost equal distribution of the wild-type cancers and *KRAS*-mutated cancers (*P* = 0.007; Table 6).

**Table 6:** The prevalences of *BRAF* V600E and *KRAS* mutations in serrated and non-serrated adenomas and adenocarcinomas with high-level microsatellite instability (MSI-H) and microsatellite stability (MSS)/low-level microsatellite instability (MSI-L)

	Mutation	All, *N*	MSS/MSI-L, *n* (%)	MSI-H, *n* (%)	*P*-value (Fisher's exact test)
All CRC	*BRAF* V600E mutation	12	7 (58.3)	5 (41.7)	0.007
		
	*KRAS* c12/13 or c59/61 mutation	31	30 (96.8)	1 (3.2)	
		
	Wild type	31	26 (83.9)	5 (16.1)	

Serrated CRC with their matched controls	*BRAF* V600E mutation	12	7 (58.3)	5 (41.7)	0.008
		
	*KRAS* c12/13 or c59/61 mutation	29	28 (96.5)	1 (3.5)	
		
	Wild type	23	18 (78.3)	5 (21.7)	

Serrated CRC	*BRAF* V600E mutation	12	7 (58.3)	5 (41.7)	0.075
		
	*KRAS* c12/13 or c59/61 mutation	16	15 (93.8)	1 (6.2)	
		
	Wild type	6	5 (83.3)	1 (16.7)	

Non-serrated CRC	*BRAF* V600E mutation	0	0 (0)	0 (0)	0.278
		
	*KRAS* c12/13 or c59/61 mutation	15	15 (100)	0 (0)	
		
	Wild type	25	21 (84)	4 (16.0)	

Matched non-serrated CRCs	*BRAF* V600E mutation	0	0 (0)	0 (0)	0.113
		
	*KRAS* c12/13 or c59/61 mutation	13	13 (100)	0 (0)	
		
	Wild type	17	13 (76.5)	4 (23.5)	

All adenomas	*BRAF* V600E mutation	4	4 (100)	0 (0)	0.588
		
	*KRAS* c12/13 or c59/61 mutation	7	7 (100)	0 (0)	
		
	Wild type	6	5 (83.3)	1 (16.7)	

Serrated adenomas	*BRAF* V600E mutation	4	4 (100)	0 (0)	NA
		
	*KRAS* c12/13 or c59/61 mutation	7	7 (100)	0 (0)	
		
	Wild type	2	2 (100)	0 (0)	

Non-serrated adenomas	*BRAF* V600E mutation	0	0 (0)	0 (0)	NA
		
	*KRAS* c12/13 or c59/61 mutation	0	0 (0)	0 (0)	
		
	Wild type	4	3 (75)	1 (25.0)	

CRC, Colorectal carcinoma; NA, not applicable.

**Table 7:** Features of serrated (*n* = 7) and non-serrated (*n* = 4) cancers with high-level microsatellite instability with respect to cancer and family history, mutation status of *KRAS*/*BRAF*, MLH1/MSH2 immunohistochemistry, and h*MLH1* methylation

Case	Type of carcinoma	Age (years)	Family history of cancer	Other cancers in patient	Mutation status of *KRAS*/*BRAF*	hMLH1 immunohistochemistry	MSH2 immunohistochemistry	h*MLH1* methylation
1	Serrated	78	Not known	No	Wild type		−	No

2	Serrated	85	Not known	No	*BRAF* V600E	−	+	Yes

3	Serrated	84	Not known	Yes, skin	*KRAS* c59/61	−	+	Yes

4	Serrated	71	Not known	Yes, breast	*BRAF* V600E	−	+	Yes

5	Serrated	68	Yes, CRC	No	*BRAF* V600E	−	+	Yes

6	Serrated	60	Not known	No	*BRAF* V600E	+	+	Yes

7	Serrated	83	Not known	No	*BRAF* V600E	−	+	Yes

8	Non-serrated	73	Not known	No	Wild type	−	+	No

9	Non-serrated	72	Not known	No	Wild type	−	+	No

10	Non-serrated	71	Not known	No	Wild type	+	−	No

11	Non-serrated	53	Not known	Yes, breast	Wild type	−	+	No

CRC, Colorectal carcinoma.

Seven of 34 serrated adenocarcinomas showed MSI-H (20.6%), and five of them (71.4%) had a concurrent *BRAF* mutation (*P* = 0.075; Table 6). One MSI-H case was wild type for both *BRAF* and *KRAS*, and another showed a *KRAS* mutation at codon 61. *KRAS* c12/13 mutations in serrated adenocarcinomas were never accompanied by MSI-H, in contrast to 15/27 (55.6%) of MSS/MSI-L cases harbouring a *KRAS* mutation (*P* = 0.075). In non-serrated adenocarcinomas, MSI-H and *KRAS* mutations did not co-occur (*P* = 0.278). Analyses with the matched controls yielded similar results (not shown).

Promoter methylation analysis was successful in 78/101 cases for h*MLH1* and in 82/101 cases for *MGMT* (Table 1). The relationships between *BRAF* and *KRAS* mutations and h*MLH1* and *MGMT* methylation status are summarized in Table 8. *BRAF* mutations were tightly associated with h*MLH1* and *MGMT* methylation, whereas *KRAS* mutations had a negative correlation with h*MLH1* and *MGMT* methylation (Table 8). The loss of MGMT expression in immunohistochemistry was associated with the corresponding methylation of the *MGMT* gene (*P* < 0.0001; Table 9). In MSS cancers, the presence of h*MLH1* and *MGMT* methylation did not correspond to the loss of immunohistochemical expression (not shown), suggesting incomplete methylation of h*MLH1.*

**Table 8:** Mutation status of *BRAF* and *KRAS* according to the promoter methylation status of h*MLH1* and *MGMT* in serrated and non-serrated cancers

	h*MLH1* methylation				*MGMT* methylation			
		
		Yes	No		All, *N*	Yes	No	
								
	All, *N*	*n* (%)	*n* (%)	*P*-value	All, *N*	*n* (%)	*n* (%)	*P*-value
Serrated adenocarcinoma								
*BRAF* V600E	10	10 (100)	0 (0.0)	0.001	11	10 (90.9)	1 (9.1)	0.019
			
Wild-type *BRAF*	17	6 (35.3)	11 (64.7		18	8 (44.4)	10 (55.6)	

*KRAS* c12/13 or c59/61	13	5 (38.5)	8 (61.5)	0.034	14	5 (35.5)	9 (64.3)	0.005
			
Wild-type *KRAS* (all)	14	11 (78.6)	3 (21.4)		15	13 (86.7)	2 (13.3)	

All non-serrated cancers								
*BRAF* V600E	0	0	0	NA	0	0	0	NA
			
Wild-type *BRAF*	43	10 (23.3)	33 (76.7)		43	18 (41.9)	25 (58.1)	

*KRAS* c12/13 or c59/61	16	1 (6.3)	15 (93.8)	0.108	16	3 (18.8)	13 (81.3)	0.084
			
Wild-type *KRAS* (all)	25	7 (28.0)	18 (72.0)		25	13 (52.0)	12 (48.0)	

Matched non-serrated cancers								
*BRAF* V600E	0	0	0	NA	0	0	0	NA
			
Wild-type *BRAF*	28	5 (17.9)	23 (82.1)		28	14 (25.0)	14 (25.0)	

*KRAS* c12/13 or c59/61	13	1 (7.7)	12 (92.3)	0.343	15	3 (23.1)	10 (76.9)	0.014
			
Wild-type *KRAS* (all)	16	4 (25.0)	12 (75.0)		16	11 (68.8)	5 (31.2)	

NA, Not applicable.

**Table 9:** The correlation of *MGMT*/h*MLH1* promoter methylation with the immunoreaction of the corresponding proteins in all cancers, serrated cancers and their matched controls

			MGMT expression*				hMLH1 expression		
							
	Promoter methylation of *MGMT*/h*MLH1*	All, *N*	Positive	Negative	*P*-value (Fisher's exact test)	All, *N*	Positive	Negative	*P*-value (Fisher's exact test)
All cases	Unmethylated	45	45	0	<0.0001	52	48	4	0.086
				
	Unmethylated and methylated	27	21	6		25	21	4	
				
	Methylated	11	5	6		5	3	2	

Serrated CRC	Unmethylated	13	13	0	0.041	13	12	1	0.186
				
	Unmethylated and methylated	17	13	4		14	11	3	
				
	Methylated	4	2	2		4	2	2	

Matched controls	Unmethylated	16	16	0	0.013	24	21	3	0.553
				
	Unmethylated and methylated	6	4	2		4	3	1	
				
	Methylated	6	3	3		1	1	0	

For *MGMT immunoreaction, tumour tissue presenting over 10% of positive cells was considered to be positive.

CRC, Colorectal carcinoma.

Our findings fit relatively well with the molecular classification of CRC proposed by Jass^6^ (Table 10). We were able to show that Jass groups 1 and 2 definitely represent serrated adenocarcinomas, but *KRAS*-mutated cases belonging to Jass group 3 were composed of serrated and non-serrated cancers. Most Jass group 4 tumours could be classified as non-serrated, and most cases in Jass group 5 were probably Lynch syndrome cases, except for one case presenting with a typical serrated growth pattern and residual serrated adenoma (illustrated in Figure 1G,H).

**Table 10:** The distribution of serrated and non-serrated cancers according to the Jass classification

	Putative Jass group						
			
	Group 1	Group 2	Group 3	Group 4	Group 5	Unclassified	
						
	MSI-H and *BRAF* V600E mutation	MSS/MSI-L and *BRAF* V600E mutation	MSS/MSI-L and *KRAS* mutation	MSS/MSI-L, wild-type *KRAS* and wild-type *BRAF*	MSI-H, wild-type *KRAS* and wild-type *BRAF*	MSI-H and *KRAS* mutation	All
							
	*n* (%)	*n* (%)	*n* (%)	*n* (%)	*n* (%)	*n* (%)	*N*
Serrated CRC	5 (14.7)	7 (20.6)	15 (44.1)	5 (14.8)	1* (2.9)	1 (2.9)	34

Non-serrated CRC	0 (0)	0 (0)	15 (37.5)	21 (52.5)	4 (10)	0 (0)	40

*P* < 0.0001, exact contingency table. Groups 1–5 follow the original categorization of Jass,^6^ with the exception of CpG island hypermethylation status and the methylation status of hMLH1 and MGMT, which are not included in the definitions of the groups.

* Single case of serrated adenocarcinoma, bearing no evidence of hereditary non-polyposis CRC, is also illustrated in Figure 1G,H, and its clinical characteristics are shown in Table 7.

CRC, Colorectal carcinoma; MSI-H, high-level microsatellite instability; MSI-L, low-level microsatellite instability; MSS, microsatellite stability.

## Discussion

This is the first study to show that *KRAS* mutations are frequent (45%) in serrated adenocarcinomas, and that the MAPK activation resulting from either *KRAS* or *BRAF* mutations is very common (79%) in serrated adenocarcinomas. Earlier studies have claimed that *BRAF* predominates over *KRAS* in biological significance in the serrated pathway,^17^,^23^ because: (i) *BRAF* mutations are specific to serrated polyps and serrated adenocarcinoma; (ii) malignant serrated endpoints presenting with *KRAS* mutations have not been reported until now; and (iii) there has been no previous evidence that *KRAS*-mutated CRCs emerge from two separate molecular pathways.

Sporadic MSI-H cancers have been attributed to the serrated pathway.^5^,^14^,^17^,^24^,^25^ In our study, MSI-H was seen in only 20.6% of serrated adenocarcinomas. A distinct association of *BRAF* mutations with MSI-H and the methylation of h*MLH1* and *MGMT* was observed among serrated cases, thus corroborating the idea that *BRAF*-mutated CRCs (Jass groups 1 and 2) represent serrated adenocarcinomas with high accuracy,^6^,^14^,^26^ but the relatively low frequency of MSI-H cancers among serrated adenocarcinomas indicates that sporadic MSI-H colorectal cancers can be attributed only to a subset of serrated adenocarcinomas.^4^,^5^,^14^

The serrated adenocarcinoma cases presenting with a residual adenoma component undoubtedly showed *KRAS* mutations (57.1%) to be twice as frequent as *BRAF* mutations (28.6%) in serrated adenocarcinomas. Either *KRAS* or *BRAF* mutation was observed in 85.7% of these cases, and in 82.4% of the serrated adenomas. These numbers suggest that MAPK activation is central for the serrated adenocarcinoma pathway, and that many CRCs with *KRAS* mutations, MSS/MSI-L and less frequent DNA hypermethylation originate from serrated polyps.

*KRAS* mutations have generally been considered to be characteristic of Vogelstein's adenoma–carcinoma model, and the integration of *KRAS* mutation in the model was justified by the high frequency of *KRAS* mutations in CRCs. Recent, well-conducted studies based on extensive case series – carried out after the recognition of serrated adenomas – have repeatedly found that *KRAS* mutations are rare in tubular adenomas, which constitute 85–90% of colorectal non-serrated adenomas.^27^,^28^ Barry *et al.*^28^ documented a 3% frequency of *KRAS* mutations in a prospective study of 303 adenomas, most mutations being observed in sessile and tubulovillous adenomas. Only two of 259 tubular adenomas (0.8%) harboured a *KRAS* mutation. Maltzman *et al.*^27^reported a 10.6% frequency for *KRAS* mutations in tubular adenomas. The high frequency of *KRAS* mutations in serrated adenomas and serrated adenocarcinomas observed in the present study explains, in part, why *KRAS* mutations are less frequent in non-serrated adenomas but occur in abount 40% of CRCs.

The high frequency of *KRAS* mutations in serrated adenocarcinomas further strengthens the importance of the colorectal serrated pathway. The estimated 15–20% frequency of serrated adenocarcinomas is based on the frequency of sporadic *BRAF*-mutated, CIM-positive CRCs.^4^ If *KRAS*-mutated serrated adenocarcinomas were taken into account, the proportion of the serrated pathway could reach 30% of all CRCs: *KRAS* mutations are more frequent in serrated polyps than in non-serrated adenomas, being observed in up to 37% of hyperplastic polyps, in up to 60% of admixed polyps, and in up to 80% of traditional serrated adenomas.^4^,^16^ The given frequencies of 30–50% for *KRAS* mutations for all colorectal cancers and 45% for serrated adenocarcinomas allow an assumption that 15–30% of *KRAS*-mutated CRCs may evolve from serrated adenomas, if we consider that the serrated pathway represents 15–20% of colorectal cancers.^4^ A similar conclusion can be drawn on the basis of polyp demographics. If traditional serrated adenomas represent 3% and non-serrated adenomas 85% of all polyps, then the 80%*KRAS* mutation rate in traditional serrated adenomas and the 3% mutation rate in non-serrated adenomas would yield *KRAS*-mutated polyps in roughly equal numbers (2.4% and 2.6% of all polyps, respectively).

It must be emphasized that serrated adenocarcinoma has not been considered as an entity in any of the previous studies on the frequency and pathogenesis of *KRAS*-mutated CRCs.^29^–^31^ This should be kept in mind when interpreting previous data on the *KRAS* mutation rate in CRCs. The Vogelstein adenoma–carcinoma model, published in 1990, was constructed ahead of the description of serrated adenomas in the same year. Therefore, it is likely that the Vogelstein model was originally contaminated by observations on (traditional) serrated adenomas bearing *KRAS* mutations misclassified as non-serrated adenomas.

The most frequent *KRAS* mutation in codons 12/13 are c12 2G→A (31–38%), c12 2G→T (21–31%), c13 2G→A (13–21%), and c12 1G→T (7–10%).^29^–^31^ The relative proportions of specific types of *KRAS* mutations that we identified were similar to those previously described (Table 5). However, the c12 G→A transition was almost completely specific to serrated adenocarcinomas, being present in 24% (10/42) of the cases, whereas in non-serrated carcinomas it was present in only 3% (2/59) of cases (*P* = 0.001). Therefore, it is likely that many CRCs with the *KRAS* c12 G→A transversion represent serrated adenocarcinomas. Besides providing a potential genetic marker for the serrated adenocarcinomas, the specificity of the c12 G→A transition may indicate the occurrence of specific aetiological factors, such as endogenous environmental or endogenous alkylating agents.^22^,^32^ The specificity of G→A transitions to serrated adenocarcinomas justifies the analysis of specific environmental risk factors, such as smoking, as possible causative agents.

In conclusion, the cumulative 79–82% frequency of *BRAF* and *KRAS* mutations in serrated adenocarcinoma and its precursors observed in our study underlines the importance of MAPK pathway activation in the serrated pathway, and suggests that these mutations are the driver mutations in the serrated pathway. The co-occurrence of *BRAF* mutations, CIM and MSI-H represents an easily identifiable subset of serrated adenocarcinoma, and *BRAF* mutation analysis can be utilized to detect these cases. However, the high frequency of *KRAS* mutations, particularly the c12 G→A transition, in serrated neoplasms emphasizes that *KRAS* mutation is an even more important alteration in the serrated pathway. Many *KRAS*-mutated CRCs originate from serrated polyps, and complete removal and follow-up of serrated adenomas with *KRAS* mutations is therefore essential to reduce the total CRC burden. The high frequency of *KRAS* mutations in serrated adenocarcinomas also indicates that most serrated adenocarcinomas are natively insensitive to epidermal growth factor receptor-blocking therapies, which are increasingly being used to treat metastatic colorectal cancer.^33^,^34^

## Abbreviations

CIM: CpG island hypermethylation
CRC: colorectal carcinoma
MAPK: mitogen-activated protein kinase
MSI: microsatellite instability
MSI-H: high-level microsatellite instability
MSI-L: low-level microsatellite instability
MSS: microsatellite stability
PCR: polymerase chain reaction
